# Supplementary material for: The genetic interaction map of the human solute carrier superfamily
Source: Mol Syst Biol. 2025 May 12;21(6):531–59. doi: 10.1038/s44320-025-00105-5 (PMC12130552; doi:10.1038/s44320-025-00105-5)
Supplement: Supplementary file 1 — Appendix [file 44320_2025_105_MOESM1_ESM.pdf]

Appendix for "The genetic interaction map of the human solute carrier superfamily"

Table of contents

Page 2: **Appendix Figure S1** showing all genetic interactions in the Cas12a-SLCxEnzyme screen that change with the environmental conditions

Page 3: **Appendix Figure S2** showing reproducibility metrics for all performed screens.

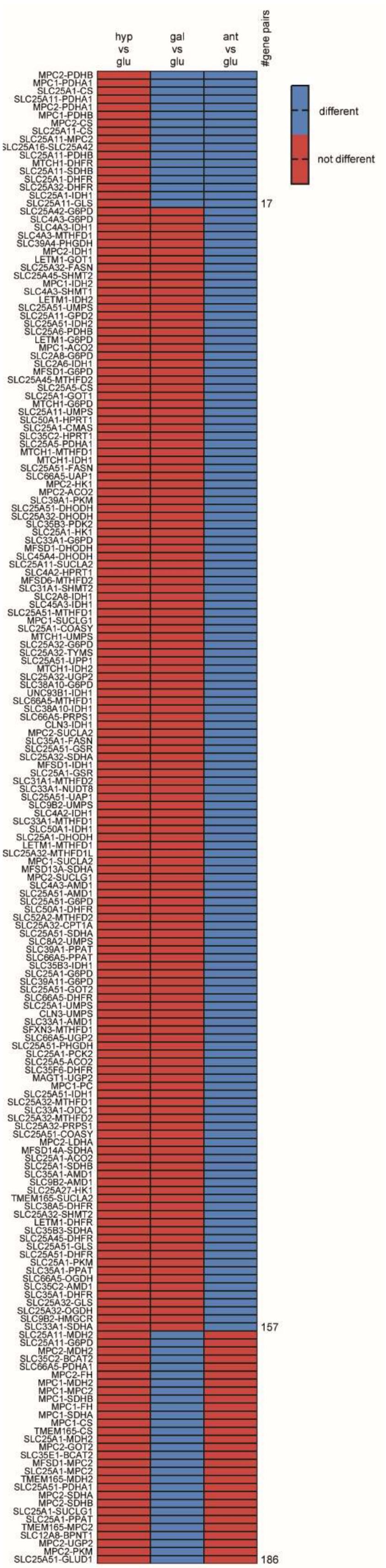

Appendix Figure S1. Heatmap showing Cas12-SLCxEnzyme genetic interactions that change in hypoxia, galactose and antimycin when compared against glucose. Based on a comparison of combined w3 and w5 dLFC values. Unpaired t test, P values were adjusted using the two-stage linear step-up procedure of Benjamini, Krieger and Yekutieli. Padj cutoff of 0.1 and |dLFC| > 0.3. 186 out of 2,357 tested genetic interactions were different in at least one condition, when compared with glucose.

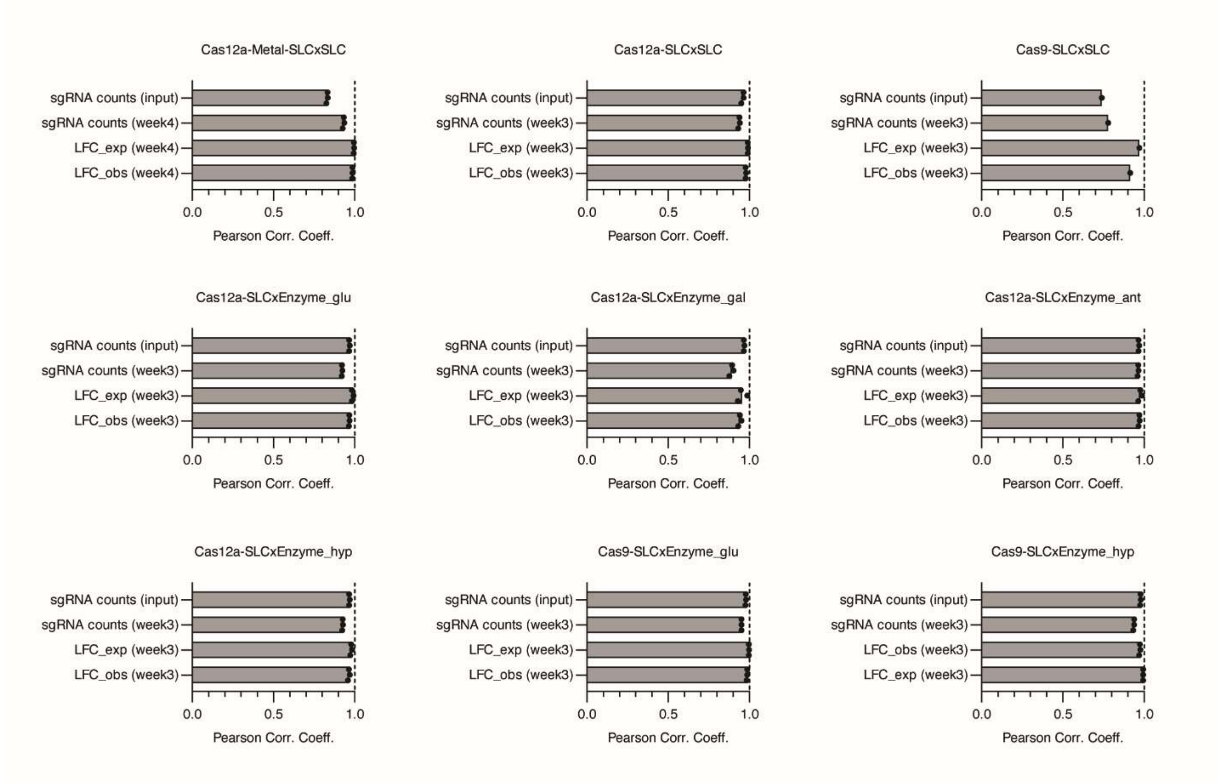

Appendix Figure S2. Reproducibility metrics of replicates for all performed screens. (A) Pearson correlation coefficients of raw sgRNA read counts, and of LFC_exp and LFC_obs at gene-level (glu: glucose, ant: antimycin, hyp: hypoxia).
