# Supplementary material for: The genetic interaction map of the human solute carrier superfamily
Source: Mol Syst Biol. 2025 May 12;21(6):531–59. doi: 10.1038/s44320-025-00105-5 (PMC12130552; doi:10.1038/s44320-025-00105-5)
Supplement: Supplementary file 11 — Expanded View Figures [file 44320_2025_105_MOESM11_ESM.pdf]

Expanded View Figures

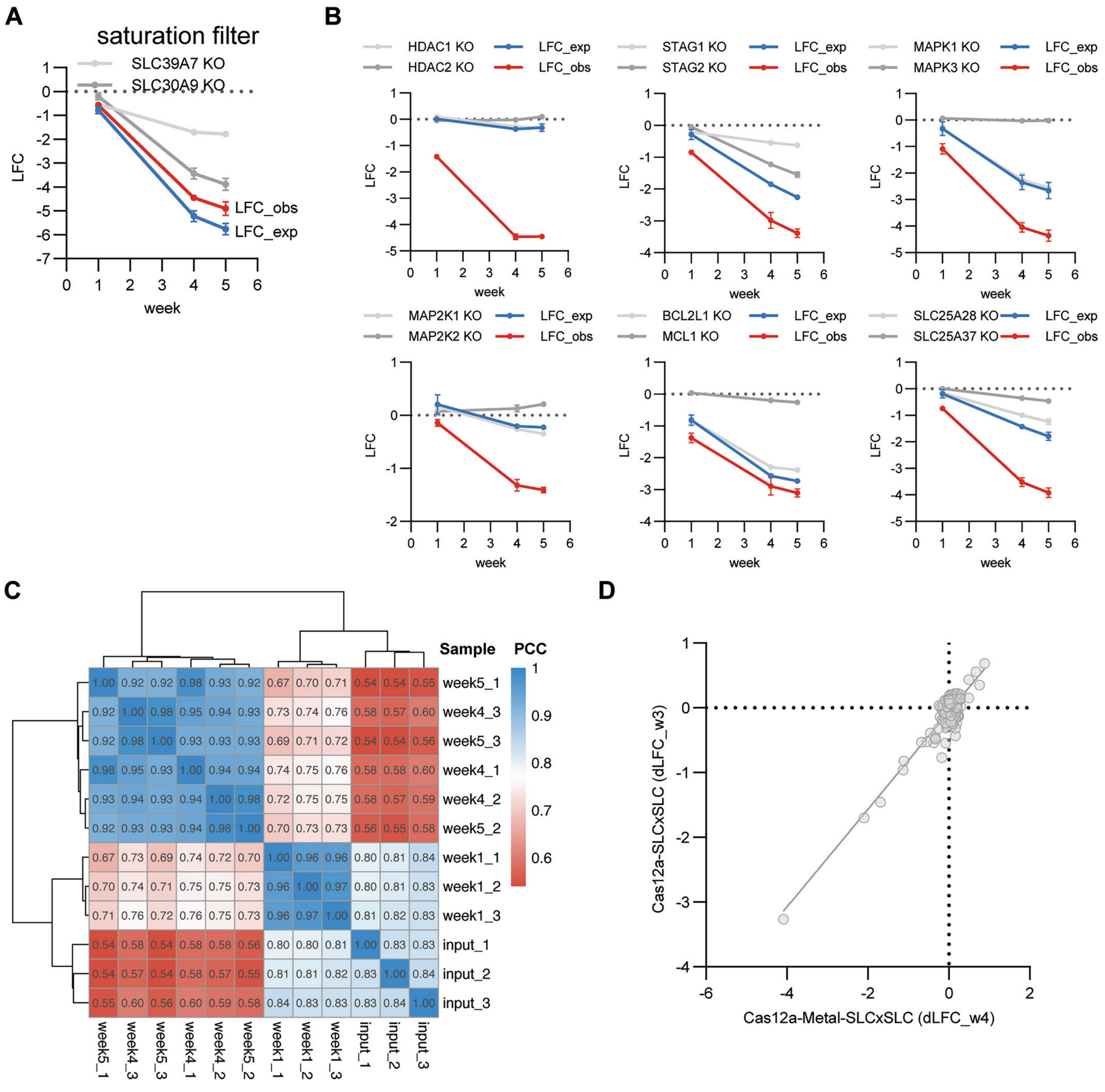

Figure EV1. Workflow for genetic interaction mapping of SLC transporters and benchmark screen targeting 21 metal SLC transporters plus controls (Cas12a-Metal-SLCxSLC).

(**A**) Example of an interaction filtered out by the saturation filter, SLC39A7-SLC30A9, because LFC_obs < (SLC30A9 KO and SLC39A7). Mean ± SD of three replicates. (**B**) Change of LFC values over time for synthetic lethal positive control pairs. Mean ± SD of three replicates. (**C**) Clustered Pearson Correlation Coefficient (PCC) matrix of raw sgRNA read counts for Cas12a-Metal-SLCxSLC replicates at different time points. (**D**) dLFC values (week 4 vs week 3) for the 210 interactions (without controls) that were part of both the Cas12a-Metal-SLCxSLC benchmark screen and the SLC superfamily-wide Cas12a-SLCxSLC screen, showing highly correlated values.

 © The Author(s)

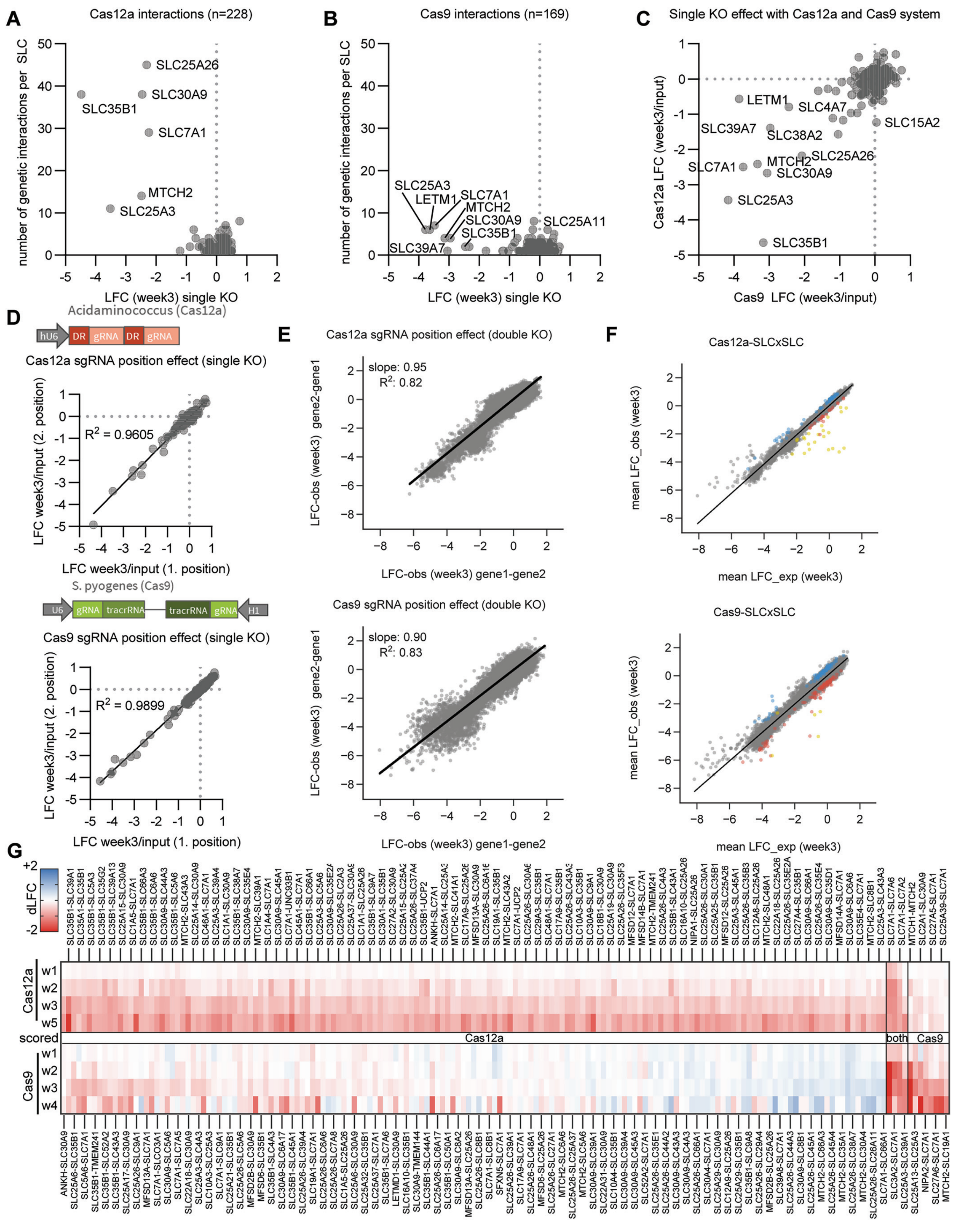
A
Cas12a interactions (n=228)
number of genetic interactions per SLC
LFC (week3) single KO
SLC25A26
SLC35B1
SLC30A9
SLC7A1
MTCH2
SLC25A3
B
Cas9 interactions (n=169)
number of genetic interactions per SLC
LFC (week3) single KO
SLC25A3
LETM1
SLC7A1
MTCH2
SLC30A9
SLC35B1
SLC25A11
SLC39A7
C
Single KO effect with Cas12a and Cas9 system
Cas12a LFC (week3/input)
Cas9 LFC (week3/input)
LETM1
SLC4A7
SLC39A7
SLC38A2
SLC15A2
SLC7A1
MTCH2
SLC25A26
SLC30A9
SLC25A3
SLC35B1
D
Acidaminococcus (Cas12a)
hU6
DR
gRNA
DR
gRNA
Cas12a sgRNA position effect (single KO)
LFC week3/input (2. position)
LFC week3/input (1. position)
R² = 0.9605
S. pyogenes (Cas9)
U6
gRNA
tracrRNA
tracrRNA
gRNA
H1
Cas9 sgRNA position effect (single KO)
LFC week3/input (2. position)
LFC week3/input (1. position)
R² = 0.9899
E
Cas12a sgRNA position effect (double KO)
slope: 0.95
R²: 0.82
LFC-obs (week3) gene2-gene1
LFC-obs (week3) gene1-gene2
Cas9 sgRNA position effect (double KO)
slope: 0.90
R²: 0.83
LFC-obs (week3) gene2-gene1
LFC-obs (week3) gene1-gene2
F
Cas12a-SLCxSLC
mean LFC_obs (week3)
mean LFC_exp (week3)
Cas9-SLCxSLC
mean LFC_obs (week3)
mean LFC_exp (week3)
G
dLFC
+2
-2
Cas12a
w1
w2
w3
w5
scored
Cas12a
both
Cas9
Cas9
w1
w2
w3
w4

◀ **Figure EV2. Genetic Interactions among SLC transporters in HCT 116 cells.**

(**A**) Number of genetic interactions per SLC in the Cas12a-SLCxSLC screen. (**B**) Number of genetic interactions per SLC in the Cas9-SLCxSLC screen. (**C**) Comparison of single KO effects between the Cas12a-SLCxSLC and Cas9-SLCxSLC screen. (**D**) Comparison of the effect of sgRNA orientation on single KO LFCs for the Cas12a-SLCxSLC and Cas9-SLCxSLC screen. (**E**) Comparison of the effect of sgRNA orientation on double KO effects for the Cas12a-SLCxSLC and Cas9-SLCxSLC screen. (**F**) Comparison of the observed double KO effect at week3 (LFC_obs (week3)) and the expected double KO effect from the sum of both single KO effects (LFC_exp (week3)). Yellow: control pairs, blue: scored synthetic viable interactions, red: scored synthetic lethal interactions. Interactions with the six most essential SLCs were not colored. (**G**) Overview of 170 interactions involving six frequently scoring SLCs that were essential as single KO in both screens based on a LFC (single KO) < −2. 158 of these interactions were identified in the Cas12a-SLCxSLC screen, while only four were found in both screens.

 © The Author(s)

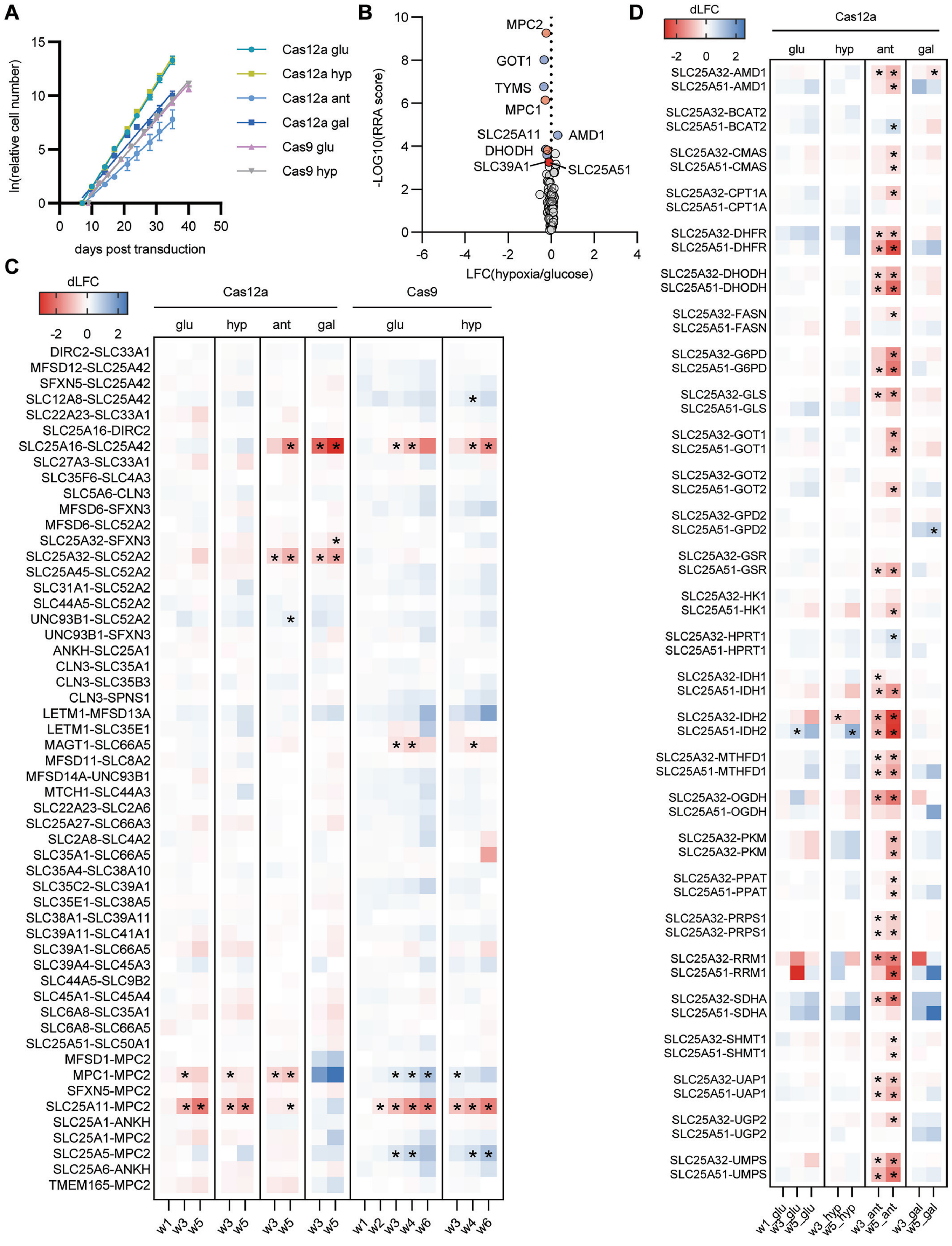

A
ln(relative cell number)
days post transduction
Cas12a glu
Cas12a hyp
Cas12a ant
Cas12a gal
Cas9 glu
Cas9 hyp
B
-LOG10(RRA score)
LFC(hypoxia/glucose)
MPC2
GOT1
TYMS
MPC1
SLC25A11
AMD1
DHODH
SLC39A1
SLC25A51
C
dLFC
Cas12a
Cas9
glu
hyp
ant
gal
D
dLFC
Cas12a
glu
hyp
ant
gal

Figure EV3. Growth rates, single knockout effects and dynamics of genetic interactions for the SLCxEnzyme screens.

(**A**) Growth rates of HCT116-Cas12a and HCT116-Cas9 cells following library transduction. Mean ± SD of three replicates. (**B**) Comparison of single KO effects in hypoxia versus glucose conditions. (**C**) Heatmap of dLFC values over time for the 54 SLC-SLC pairs additionally included in the SLCxEnzyme library. Stars indicate significance (padj < 0.1 in all three replicates). (**D**) Heatmap of dLFC values over time for SLC25A32 and SLC25A51 interactions. Displayed are all interactions that were significant for either SLC25A32 or SLC25A51. Stars indicate significance (padj < 0.1 in all three replicates).

© The Author(s)

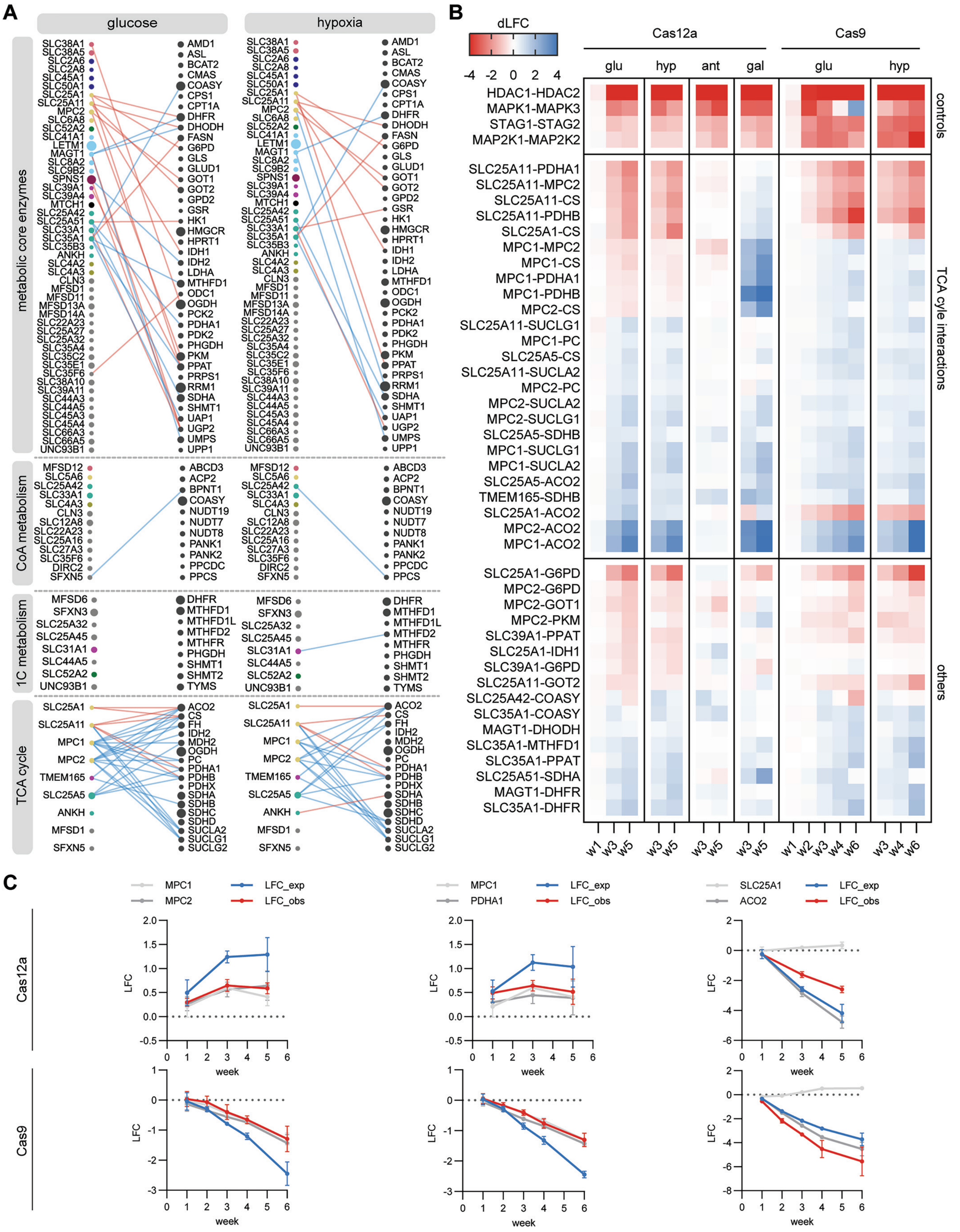
A
glucose
hypoxia
metabolic core enzymes
CoA metabolism
1C metabolism
TCA cycle
SLC38A1
SLC38A5
SLC2A6
SLC2A8
SLC45A1
SLC50A1
SLC25A1
SLC25A11
MPC2
SLC6A8
SLC52A2
SLC41A1
LETM1
MAGT1
SLC8A2
SLC9B2
SPNS1
SLC39A1
SLC39A4
MTCH1
SLC25A42
SLC25A51
SLC33A1
SLC35A1
SLC35B3
ANKH
SLC4A2
SLC4A3
CLN3
MFSD1
MFSD11
MFSD13A
MFSD14A
SLC22A23
SLC25A27
SLC25A32
SLC35A4
SLC35C2
SLC35E1
SLC35F6
SLC38A10
SLC39A11
SLC44A3
SLC44A5
SLC45A3
SLC45A4
SLC66A3
SLC66A5
UNC93B1
AMD1
ASL
BCAT2
CMAS
COASY
CPS1
CPT1A
DHFR
DHODH
FASN
G6PD
GLS
GLUD1
GOT1
GOT2
GPD2
GSR
HK1
HMGCR
HPRT1
IDH1
IDH2
LDHA
MTHFD1
ODC1
OGDH
PCK2
PDHA1
PDK2
PHGDH
PKM
PPAT
PRPS1
RRM1
SDHA
SHMT1
UAP1
UGP2
UMPS
UPP1
MFSD12
SLC5A6
SLC25A42
SLC33A1
SLC4A3
CLN3
SLC12A8
SLC22A23
SLC25A16
SLC27A3
SLC35F6
DIRC2
SFXN5
ABCD3
ACP2
BPNT1
COASY
NUDT19
NUDT7
NUDT8
PANK1
PANK2
PPCDC
PPCS
MFSD6
SFXN3
SLC25A32
SLC25A45
SLC31A1
SLC44A5
SLC52A2
UNC93B1
DHFR
MTHFD1
MTHFD1L
MTHFD2
MTHFR
PHGDH
SHMT1
SHMT2
TYMS
SLC25A1
SLC25A11
MPC1
MPC2
TMEM165
SLC25A5
ANKH
MFSD1
SFXN5
ACO2
CS
FH
IDH2
MDH2
OGDH
PC
PDHA1
PDHB
PDHX
SDHA
SDHB
SDHC
SDHD
SUCLA2
SUCLG1
SUCLG2
B
dLFC
-4 -2 0 2 4
Cas12a
Cas9
glu hyp ant gal
glu hyp
controls
TCA cyle interactions
others
HDAC1-HDAC2
MAPK1-MAPK3
STAG1-STAG2
MAP2K1-MAP2K2
SLC25A11-PDHA1
SLC25A11-MPC2
SLC25A11-CS
SLC25A11-PDHB
SLC25A1-CS
MPC1-MPC2
MPC1-CS
MPC1-PDHA1
MPC1-PDHB
MPC2-CS
SLC25A11-SUCLG1
MPC1-PC
SLC25A5-CS
SLC25A11-SUCLA2
MPC2-PC
MPC2-SUCLA2
MPC2-SUCLG1
SLC25A5-SDHB
MPC1-SUCLG1
MPC1-SUCLA2
SLC25A5-ACO2
TMEM165-SDHB
SLC25A1-ACO2
MPC2-ACO2
MPC1-ACO2
SLC25A1-G6PD
MPC2-G6PD
MPC2-GOT1
MPC2-PKM
SLC39A1-PPAT
SLC25A1-IDH1
SLC39A1-G6PD
SLC25A11-GOT2
SLC25A42-COASY
SLC35A1-COASY
MAGT1-DHODH
SLC35A1-MTHFD1
SLC35A1-PPAT
SLC25A51-SDHA
MAGT1-DHFR
SLC35A1-DHFR
w1 w3 w5 w3 w5 w3 w5 w3 w5 w1 w2 w3 w4 w6 w3 w4 w6
C
MPC1 MPC2 LFC_exp LFC_obs
MPC1 PDHA1 LFC_exp LFC_obs
SLC25A1 ACO2 LFC_exp LFC_obs
Cas12a
Cas9
LFC
week

Figure EV4. Comparison of SLCxEnzyme screens using CRISPR-Cas9 and CRISPR-Cas12a systems.

(**A**) Orthogonal CRISPR-Cas9 screen testing the same gene pairs as shown in Fig. 3D but only in two growth conditions. (**B**) Side-by-side time course dLFC values genetic interactions that were detected in either the Cas12a-SLCxEnzyme, the Cas9-SLCxEnzyme or both screens. (**C**) Some interactions are buffering in both Cas12a and Cas9 but in opposite directions, likely due to clonal differences between the HCT116-Cas12a and -Cas9 clones used (e.g., MPC1-MPC2 and MPC1-PDHA). This results in the same genetic interaction classification (lethal vs viable) while exhibiting opposite growth phenotypes in the Cas12a- vs the Cas9- clone. A few interactions disagreed in classification, e.g., SLC25A1-ACO2. Mean ± SD of three replicates.

 © The Author(s)

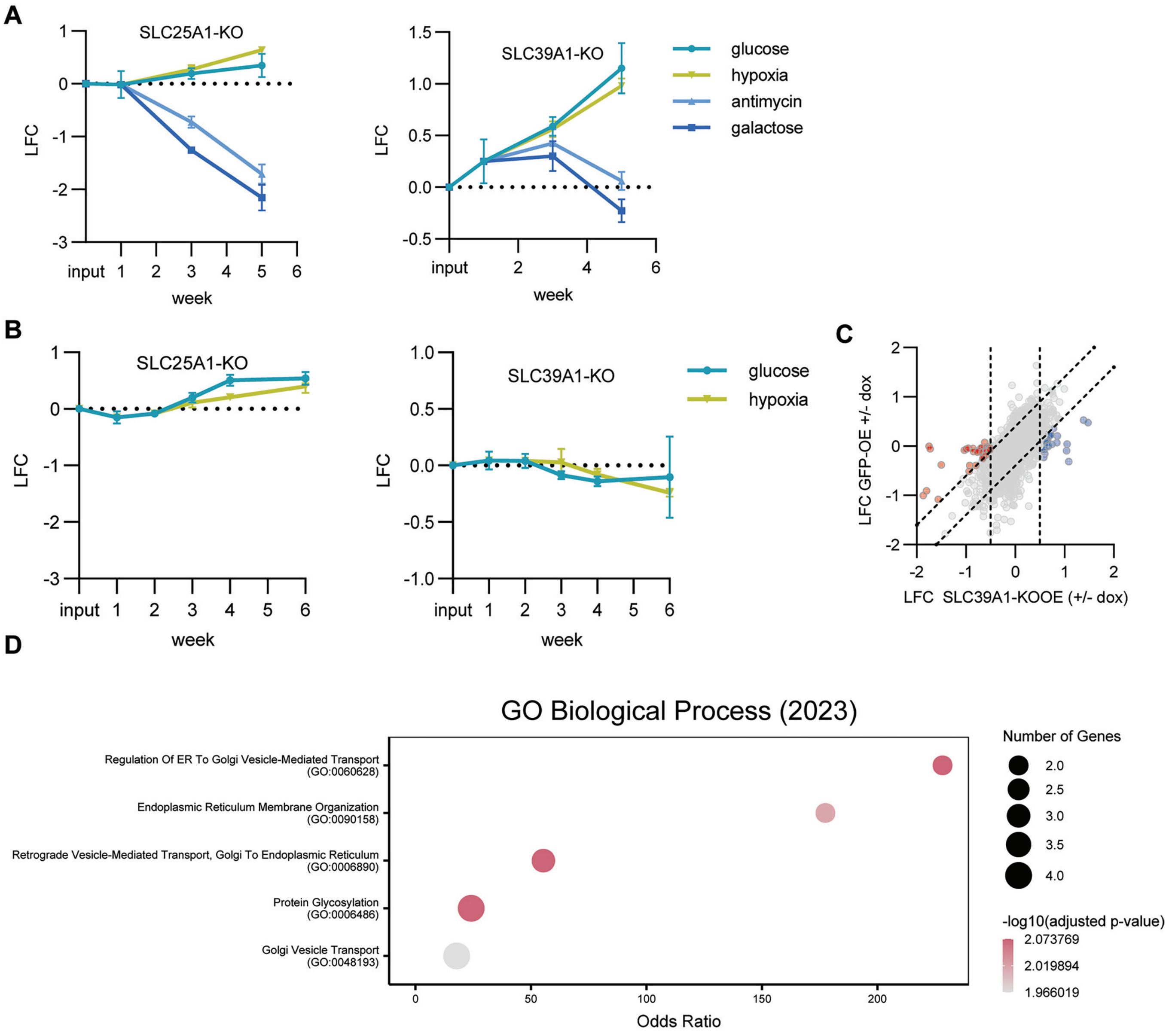

Figure EV5. Genetic interactions and transcriptomic changes of SLC39A1 and SLC25A1.

(**A**) Single KO effects of SLC25A1 and SLC39A1 across different growth conditions in the Cas12a-SLCxEnzyme screen. Mean ± SD of three replicates. (**B**) Single KO effects of SLC25A1 and SLC39A1 across different growth conditions in the Cas9-SLCxEnzyme screen. Mean ± SD of three replicates. (**C**) Transcriptomics changes in HCT116-SLC39A1-KO-OE cells after doxycycline-induced expression compared against GFP-OE control, for exclusion of effects caused by doxycycline incubation. (**D**) Gene ontology enrichment of the protein-protein interaction network shown in Fig. 5D. Significance was determined using Fisher's exact test and *p* values were adjusted for multiple testing using the Benjamini-Hochberg method.

© The Author(s)
